# Supplementary material for: Causal Relationship between Aging and Anorexia Nervosa: A White-Matter-Microstructure-Mediated Mendelian Randomization Analysis
Source: Biomedicines. 2024 Aug 16;12(8):1874. doi: 10.3390/biomedicines12081874 (PMC11351342; doi:10.3390/biomedicines12081874)
Supplement: Supplementary file 1 [file biomedicines-12-01874-s001.zip › Supplementary Figure/Figure S8/Leaveoneout plot of MR analysis for TL to ubm-b-2004.pdf]

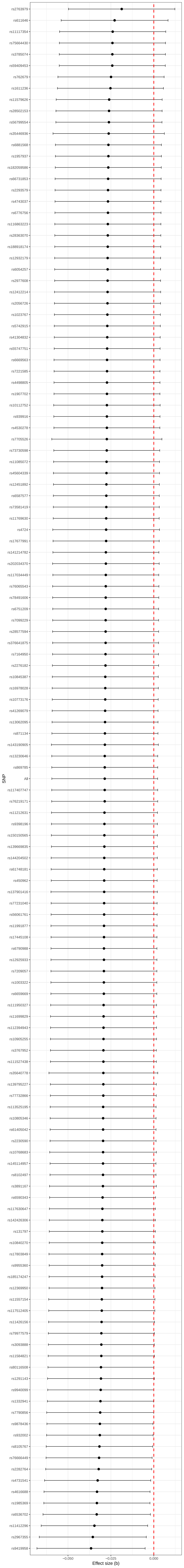
rs2763979
rs611646
rs11117354
rs75664430
rs3785074
rs59409453
rs762679
rs1611236
rs11579626
rs28502153
rs56799554
rs35446936
rs6881568
rs1957937
rs182059586
rs66731853
rs2293579
rs4743037
rs6776756
rs116863223
rs28363070
rs188918174
rs12932179
rs6054257
rs2977608
rs12412214
rs2056726
rs1023767
rs5742915
rs41304832
rs55747751
rs6669563
rs7221585
rs4498805
rs1907702
rs10112752
rs939916
rs4530278
rs7705526
rs73730598
rs11085072
rs45604339
rs12451892
rs6587577
rs73581419
rs11769630
rs4724
rs17677991
rs141214782
rs202034370
rs117034449
rs76065543
rs78491606
rs6751209
rs7099229
rs28577594
rs376641875
rs7164950
rs2276182
rs10845387
rs16978028
rs10773176
rs41269079
rs13062095
rs871134
rs143190905
rs13230646
rs869785
All
rs117407747
rs76219171
rs11212631
rs9398196
rs150150565
rs139669835
rs144204502
rs61748181
rs450962
rs137901416
rs77231040
rs56061761
rs11991877
rs17445108
rs6790988
rs12925933
rs7209057
rs1003322
rs6659669
rs111950327
rs11699829
rs112394943
rs10905255
rs3767952
rs111527438
rs35640778
rs139795227
rs77732866
rs113525195
rs10805346
rs61405042
rs2230590
rs10768683
rs145114957
rs8102497
rs3891167
rs6590343
rs117630647
rs142426306
rs131797
rs10840270
rs17803849
rs9955360
rs185174247
rs12369950
rs11557154
rs117512405
rs11426156
rs79977579
rs3093888
rs11584821
rs80116508
rs1291143
rs9940099
rs1332941
rs7790856
rs9878436
rs932002
rs8105767
rs76666449
rs2282764
rs4731541
rs4618688
rs1985369
rs6536702
rs11412296
rs2967355
rs9419958
SNP
-0.050
-0.025
0.000
Effect size (b)
